# A case report of sustained remission after radiotherapy combined with ICI in NEPC with primary drug resistance to chemotherapy

**DOI:** 10.3389/fonc.2024.1360956

**Published:** 2024-04-26

**Authors:** Tengfei Li, Yanan Wang, Yueqiang Jiang, Zixi Wu, Zhiquan Hu, Zhihua Wang, Chunguang Yang

**Affiliations:** ^1^ Department of Urology, Tongji Hospital Affiliated Tongji Medical College of Huazhong University of Science and Technology (HUST), Wuhan, China; ^2^ Department of Geriatrics, Tongji Hospital, Tongji Medical College Huazhong University of Science and Technology, Wuhan, China

**Keywords:** prostate cancer, NEPC, immune checkpoint inhibitor, radiotherapy, drug resistance

## Abstract

Advanced prostate cancer (PCa) is usually treated initially with androgen deprivation therapy (ADT). Although they experience a period of disease regression, most patients progress to metastatic castration-resistant prostate cancer (mCRPC). Patients with mCRPC now have an unprecedented number of approved treatment options, including chemotherapies, hormone therapies, targeted therapies, etc. However, the improvement of overall survival (OS) in patients with mCRPC and its special subtype neuroendocrine prostate cancer (NEPC) is limited. In recent years, with the use of immune checkpoint inhibitors (ICIs), such as PD1/PDL1 and CTLA4 inhibitors, immunotherapy has once again become a promising treatment choice to stimulate antitumor immunity. However, the efficacy of NEPC receiving ICI has not been reported. Here, we describe a patient with mCRPC who developed primary resistance to current endocrine and chemotherapy regimens and progressed to mCRPC with NEPC as the main component, showing a significant and lasting response to PD1 monoclonal antibody combined with radiotherapy.

## Background

In recent years, with the extension of life expectancy, the aging of population, the change of lifestyle and the popularization and application of prostate cancer screening methods, such as prostate specific antigen (PSA), the incidence of prostate cancer is increasing rapidly (1, 2). In 2015, There were 72,000 new cases of prostate cancer, and the incidence rate was 10.23/100,000, ranking sixth among male malignant tumors in China. There were 31,000 deaths with the mortality rate of 4.36/100,000, and ranks 10th in male malignant tumors (2). Current first-line treatments that extend survival for mCRPC include abiraterone, enzalutamide, docetaxel and sipuleucel-T (3). However, these treatments only extend the OS for a few months, many patients eventually develop drug resistance, and the tumor progresses (4, 5). Moreover, due to the extensive use of these drugs, the occurrence of an AR-independent, more malignant and lethal neuroendocrine prostate cancer (NEPC) is rapidly increasing, accounting for 25% of metastatic CRPC patients (6). Patients with NEPC have a local invasion or distant metastasis, and the prognosis is very poor with a survival time of less than 1 year. At present, the biological behavior of NEPC is not well explored, the mechanism of neuroendocrine transdifferentiation in CRPC is not clear, and the clinical treatment for NEPC is limited (7, 8).

In 2015, the KEYNOTE-016 trial identified MSI-H/dMMR as a biomarker indicating the effectiveness of immunotherapy and pointed out the potential use of ICIs. In 2017, the FDA approved two types of ICIs (pembrolizumab and nivolumab) for adult and pediatric patients with unresectable or metastatic, microsatellite instability-high (MSI-H) or mismatch repair deficient (dMMR) solid tumors, including PCa (9). A phase II trial of 258 mCRPC patients treated with pembrolizumab showed an objective response rate of approximately 4%, but these responses were long-lasting (3). Combined immunotherapy is being studied, including KEYNOTE-199, KEYNOTE-365 test, KEYNOTE-641 and KEYNOTE-921 test and so on. However, the regimen and efficacy of pembrolizumab in patients with MSI-H CRPC with neuroendocrine differentiation are not clear. Few patients with NEPC were included in these preliminary studies. Here, we report a case of CRPC with NEPC as the main component of primary resistance to new endocrine and chemotherapeutic drugs. The patient received PD1 combined with radiotherapy and showed lasting benefits.

## Case presentation

This case report outlines the treatment of a primary extensively drug-resistant CRPC patient with NEPC as the main component, which is still being treated thus far. The treatment regimen combined with radiotherapy and PD1 has achieved lasting benefits.

In September 2021, a 69-year-old man was admitted to our hospital with intermittent gross hematuria with frequent urination for one week. Prostate biopsy and MR analysis revealed prostate cancer (Gleason score 5 + 5 = 10), breaking through the capsule, invading both seminal vesicle glands and bladder, blurring the boundary between local and anterior rectal wall, and increasing and enlarged pelvic lymph nodes (prostate specific antigen, PSA 4.442 ng/mL). He is a newly diagnosed prostate cancer patient who has not previously received any endocrine therapy or chemotherapy. The results of gene detection showed MSH2 and MSH6 deletion (dMMR), high MSI-H, high tumor mutation load (TMB-H) and BRCA2 mutation. In this case, we suspected that the patient had rectal invasion, and after introducing the surgical plan to the patient, he refused the operation.

**Figure 1:**
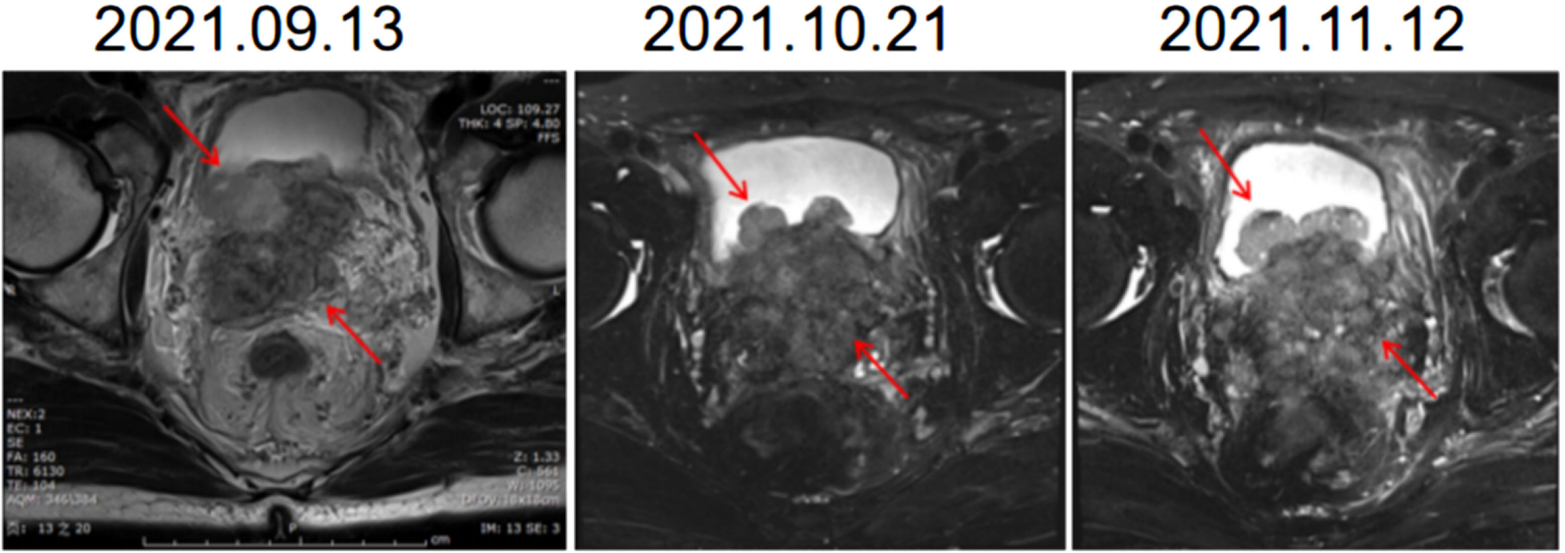
Images of MR during endocrine therapy combined with PARPi therapy. The shape of the prostate is abnormal, breaking through the prostate capsule, involving bilateral seminal vesicle glands, protruding into the bladder and growing. MR, Magnetic resonance.

Initially, we arranged for the patient to receive PARP inhibitors combined with endocrine therapy (triptoreline+ abiraterone + prednisone). Within two months after the start of treatment, the frequency of urination was slightly improved, and the gross hematuria disappeared, but the tumor continued to show imaging progress (**Figure 1**). Although the PSA value decreased from 4.442 ng/mL to 0.048 ng/mL, the value of neuron-specific enolase (NSE) increased during the monitoring process.

**Figure 2:**
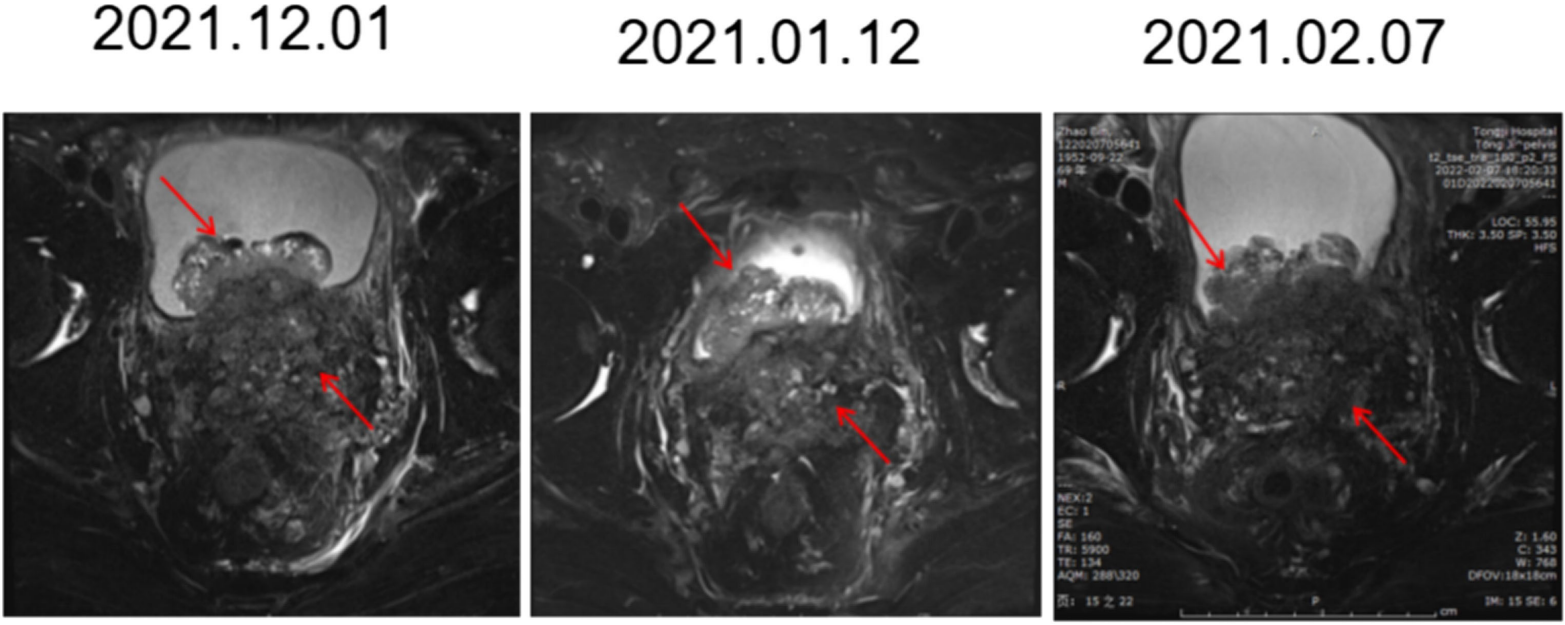
Images of MR in chemotherapy (2 cycles of docetaxel and one cycle of EP). The prostate mass broke through the capsule and involved both the seminal vesicle glands and bladder. Compared with the front, it gradually became fuller and progressed over time.

Prostate biopsy was performed again to evaluate the progressive tumor tissue, to determine whether there were neuroendocrine components and to apply for consultation with pathologists at home and abroad. After multidisciplinary, multihospital expert center consultation and discussion, the diagnosis was castration-resistant mixed carcinoma of the prostate (adenocarcinoma + small cell carcinoma) with NEPC as the main component (**Supplementary Figure**). The treatment plan was adjusted, and two cycles of docetaxel chemotherapy and one cycle of Etoposide-cisplatin (EP) chemotherapy were arranged for the patient. However, the patient’s condition progressed in a very short period of time. The patient urinates frequently, urinates urgently, and urination pain is aggravated. Imaging examination showed that the patient gradually developed bilateral hydronephrosis and bilateral ureteral dilatation, and continued imaging progression of the tumor (**Figure 2**). To protect renal function, right nephrostomy was performed at the fifth month after admission. During the whole course of chemotherapy, PSA remained at a low level but showed an upwards trend after a short period of decline. Similarly, NSE rose again after a short-term decline.

Due to his own hypertension grade 2 (very high risk group), right thyroid nodules (TI-RADS4a class), primary hyperparathyroidism and hyperlipidemia, the patient was not suitable for operation. We request national consultation to guide the next step of local radiotherapy and systemic immunotherapy.

## Treatment course

**Figure 3:**
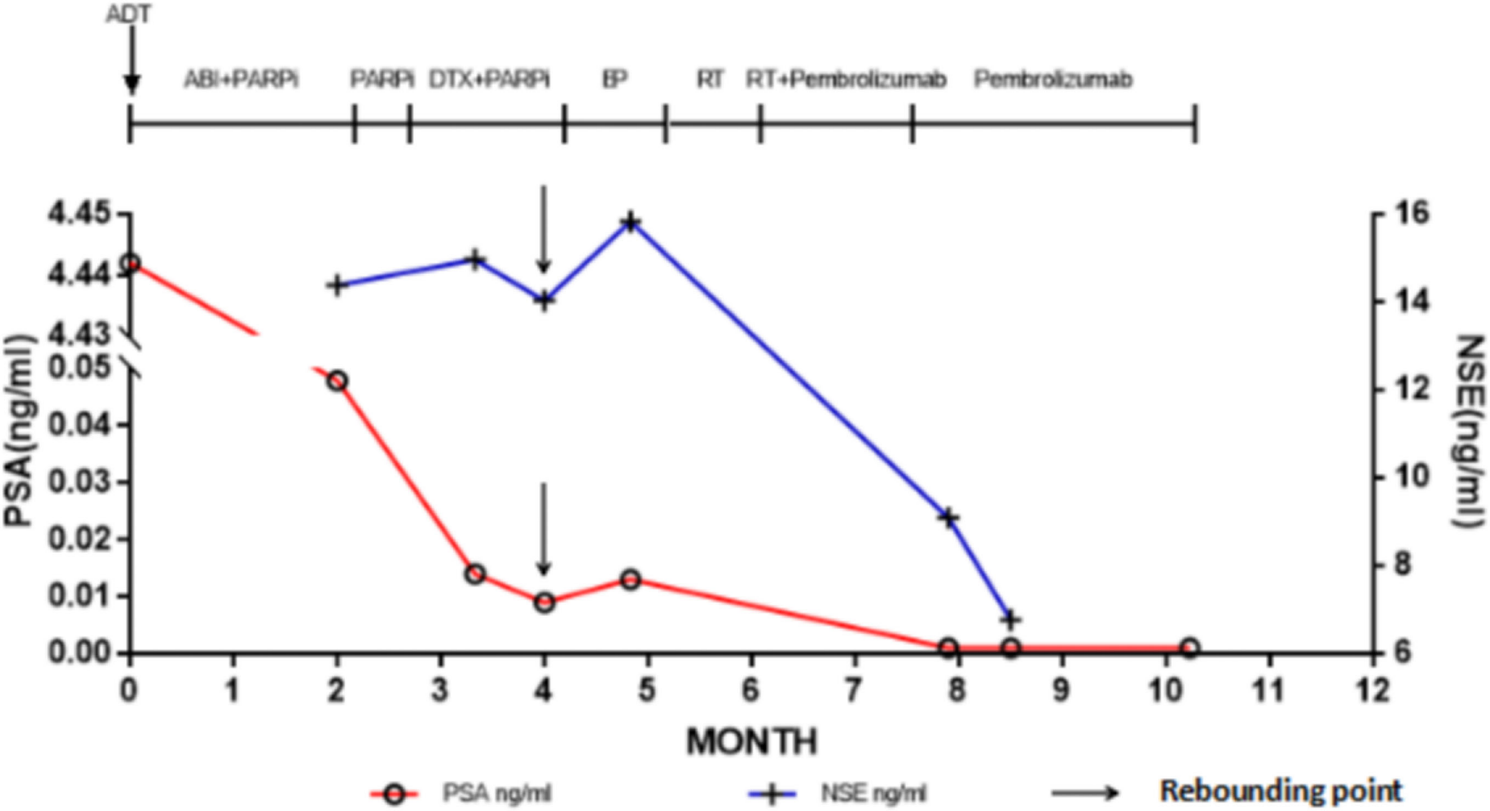
Prostate-specific antigen values along the patient’s clinical course. The point indicated by the arrow is the turning point of the rebound of the marker. ADT, Androgen deprivation therapy; ABI, Abiraterone; DTX, Docetaxel; NSE, Neuron-specific-enolase; PARPi, Poly adenosine diphosphate ribose polymerase inhibitor; PSA, Prostate-specific antigen; RT, Radiotherapy.

**Figure 4:**
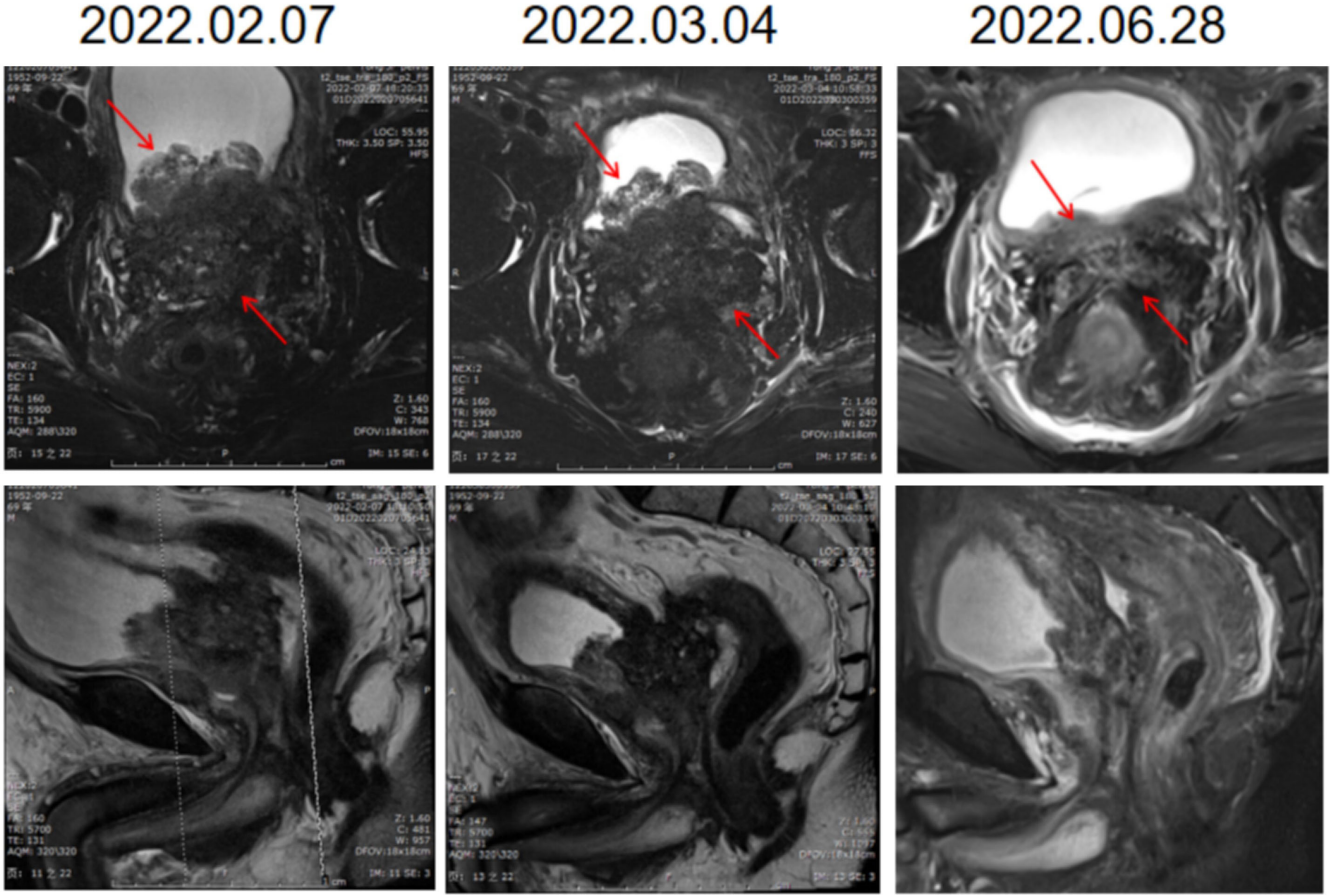
Imaging findings of local pelvic radiotherapy combined with PD1. The first line of the pictures is coronal and the second line is sagittal. Each column is an image of the prostate on the same date. The local size of the prostate tumor was significantly smaller after treatment.

Six days after right nephrostomy, the patient received pelvic local radiotherapy: DtGTV 50 Gy/25 F, CTV 45 Gy/25 F on the basis of maintaining ADT. Pembrolizumab would be used on March 16, 2022 (200 mg Magna Q3w). After the second cycle of pembrolizumab treatment, the local size of the prostate tumor was significantly reduced. Therefore, on May 11, 2022, double J ureteral tubes were implanted, and right nephrostomy tubes and catheters were pulled out. In the follow-up process, the urination of the patient was unobstructed, and the urinary tract irritation gradually improved. After the third cycle, the PSA value decreased to an undetectable level (< 0.008 ng/mL), and the NSE value continued to decline to a low level during the treatment but did not rise. The changes in PSA and NSE levels along the patient’s clinical course after admission for treatment are summarized in **Figure 3**. The imaging results also showed that the local PCa lesions and retroperitoneal lymph nodes were significantly smaller than those before the beginning of pembrolizumab treatment (**Figure 4**). Radiotherapy combined with PD1 therapy has brought lasting benefits to patients for up to 5 months, but it is still in maintenance therapy, and there is no further progress.

## Discussion

ADT is the most widely used basic treatment for patients with advanced metastatic prostate cancer (Moca). In recent years, the emergence of a variety of new endocrine drugs, such as abiraterone, enzalutamide, apalutamide and darolutamide, has also provided more options for ADT of PCa. Several clinical studies have shown that castration alone combined with abiraterone can prolong progression-free survival and gain survival benefits in patients with mPCa (10–12). At the same time, PARP inhibitors have been proven to be effective in CRPC patients with homologous recombination repair (HRR) gene mutations such as BRCA1/2 (13). In this case, genetic detection revealed MSH2 and MSH6 deletion (dMMR) as well as BRCA2 mutation. Significant imaging progress was observed during ADT, abiraterone combined with prednisone and PARP inhibitors. However, the detection of tumor markers in this patient, as shown in **Figure 1**, showed that PSA significantly decreased at a low level (< 0.1 ng/ml), and NSE showed an upwards trend. Wang et al. conducted a systematic retrospective analysis of 123 patients with NEPC to assess the risk factors associated with the development and survival of NEPC (14). The median time from initial diagnosis to progression of prostate cancer was 20 months. A high Gleason score (≥ 8) at diagnosis was an independent risk factor for early prostate cancer progression (HR, 1.66; P=0.032). At the same time, for patients with particularly invasive, atypical spread and/or progression of the disease and with low or no elevated PSA levels, suspected NEPC transformation and metastasis biopsy can also be considered (15). NEPC has shown sensitivity to platinum drugs, and patients with small cell cancer can consider platinum-based chemotherapy (16, 17). In this case, the patient underwent another biopsy, and the result was diagnosed as castrated resistant mixed prostate cancer with NEPC as the main component after consultation and discussion in the expert center of multiple disciplines and multiple hospitals. The patient had not received chemotherapy in the past and then received docetaxel chemotherapy; EP chemotherapy had no obvious effect, and the disease continued to progress. For prostate cancer patients, after long-term first-line treatment, drug resistance will eventually develop and develop into CRPC. However, in this case, the patient had never received other related treatment before and did not respond to the commonly used first-line prostate cancer treatment, showing drug resistance and rapid progression of the disease. The condition that occurred during the treatment of this patient is rare. Turina et al. reported a patient with PCa with changes in BRCA2 who had a significant response to ADT plus enzalutamide for 4 months, followed by NEPC with a complete response to etoposide/carboplatin and continued remission after 9 months of treatment with oxalapril (18). Pandya et al. reported another case of NEPC with BRCA2 changes 12 months after treatment with ADT and abiraterone combined with prednisone. NEPC appeared 16 months after treatment, which showed a stable effect for 6 months in etoposide/carboplatin chemotherapy (19). In this case, the patient had no significant effect during the 2 months of treatment with ADT, abiraterone combined with prednisone and PARP inhibitor, as well as in the subsequent chemotherapy regimen, showing continuous imaging progress. To the best of our knowledge, this is the first case of PCa that has shown extensive primary drug resistance to first-line treatment.

The emergence of immunotherapy has completely changed the treatment pattern of several blood and solid malignant tumors, especially in patients with detection of MSI-H and TMB-H, reporting unprecedented response rates. Examples will be given in later sections. MMR can repair the insertion or deletion of microsatellite sequences. The MMR system includes four main proteins, MLH1, MSH2, MSH6 and PMS2. When a mismatch is detected, MSH2, MSH6,MLH1 and PMS2 form a heterodimer and repair the mismatch (20). In normal cells, the four main proteins of MMR system are expressed normally, and the mismatch repair system is intact, which is called pMMR. In tumor cells, the loss of expression of any one of the four proteins in the MMR system can lead to the defect of mismatch repair function, which is called dMMR. DNA mismatch repair gene mutation will lead to the loss of the corresponding repair protein function, thus affecting the mismatch repair function of DNA. DNA replication leads to the accumulation of a large number of mismatch, which leads to microsatellite instability, that is, the occurrence of MSI. DMMR is equivalent to MSI-H phenotype. Therefore, clinical results detected as dMMR can also benefit from immunotherapy (21). First of all, because microsatellite instability means that there are a large number of gene mutations in the body, these mutated genes will form new proteins through transcription and translation, which are new antigens for tumor microenvironment. the new antigen has a high degree of immunogenicity, which can improve the immune response of tumor infiltrating lymphocytes (22). In addition, because MSI-H tumors are usually accompanied by TMB-H, and high TMB is also one of the signs of sensitivity to immunotherapy. High TMB means that there are a large number of gene mutations in the body, the more gene mutations, the easier it is to form new antigens, the easier it is to cause immune response (23). At present, there have been related clinical trials in PCa patients, and some cases have shown that PD1 has practical clinical significance. Manogue et al. reported a patient with mCRPC who was in complete remission after being treated with pembrolizumab. The patient showed changes in MSH2 by tissue sampling (24). Ravindranathan et al. reported two mCRPC patients with MSI-H status. After receiving pembrolizumab treatment, a stable and lasting effect was obtained, resulting in an excellent clinical response measured with liquid biopsies before and after initiation of therapy, which demonstrated a significant reduction in somatic-variant allele frequency in addition to a decrease in prostate serum antigen levels (25). Shimizu et al. reported that the size of lymph node metastasis and local PCa lesions decreased significantly in an MSI-H mCRPC patient treated with pembrolizumab, and the PSA value remained at an undetectable level 18 months after the start of treatment (26). KEYNOTE-199 studies that enrolling mCRPC patients with RECIST-measurable PD-L1-positive, PD-L1-negative disease and bone-predominant disease, respectively, have confirmed the preliminary efficacy of PD1 in patients with CRPC and HRR gene mutations (27). However, there is still a lack of known effective treatments for refractory NEPC that are ineffective to chemotherapy (19). Although there are also case reports about the long-term and stable efficacy of PD-1 monotherapy in the treatment of NEPC, the effective rate of PD-1 monotherapy for overall prostate cancer patients is still at a very low level (28). How to transform cold-type prostate cancer into hot prostate cancer and make PD-1 suitable for more prostate cancer patients has become a clinical problem.In this case, the patient received local pelvic radiotherapy combined with PD1 and showed a significant effect. At 5 months after the treatment of local pelvic radiotherapy combined with PD1 compared with before treatment, the volume of prostate tumor was significantly reduced and the disease is no longer progressing.

At the same time, after receiving PD1 combined with radiotherapy, the retroperitoneal lymph nodes outside the area of local radiotherapy also shrank. Radiotherapy stimulates the release of tumor-associated antigens, activation of T cells and the inflammatory microenvironment, transforming cold tumors into hot tumors and leading to a systemic antitumor host immune response. The combination of radiation and ICI therapy can enhance the induction of the immune response to radiation-targeted disease areas and the whole body (29). The result of this combination of radiotherapy and ICI therapy is called the abscopal effect. Radiotherapy has been shown to have a synergistic effect with ICIs in patients with mCRPC (29). Han et al. also reported imaging evidence of a reduced prostate mass and no rectal wall involvement after PD1 combined with radiotherapy in a patient with rectal invasion of mCRPC (30). It is not difficult to see that PD1 has a significant and lasting effect on some patients who are found to have MSI-H or dMMR based on gene sequencing. In this case, pembrolizumab combined with radiotherapy showed an unexpected response to advanced CRPC with NEPC as the main component of first-line treatment. We are still tracking the follow-up, and the patient is in good condition now. Our findings provide new ideas and support for the treatment of NEPC. However, in this case, if the treatment method can be determined earlier, the pain and injury of the patient may be reduced during the treatment. Further exploration is needed to determine the timing and criteria of radiotherapy combined with PD-1.

## Conclusion

Here, we report a patient with mCRPC who was resistant to endocrine therapy and chemotherapy. After using various first-line treatments that are ineffective and progress to mCRPC with NEPC as the main component, we report a case of long-term benefit from PD1 combined with radiotherapy. It provides a new method and support for improving the effective rate of PD-1 in the treatment of patients with prostate cancer. Further studies are needed to determine the population of prostate cancer patients who are suitable for PD1 combined with radiotherapy.

## Data availability statement

The original contributions presented in the study are included in the article/**Supplementary Material**. Further inquiries can be directed to the corresponding authors.

## Ethics statement

The studies involving humans were approved by Medical Ethics Committee of Tongji Hospital. The studies were conducted in accordance with the local legislation and institutional requirements. The participants provided their written informed consent to participate in this study. Written informed consent was obtained from the individual(s) for the publication of any potentially identifiable images or data included in this article.

## Author contributions

TL: Writing – original draft, Writing – review & editing, Data curation, Formal analysis, Investigation, Visualization. CY: Resources, Supervision, Writing – review & editing. YW: Data curation, Resources, Supervision, Writing – review & editing. YJ: Project administration, Resources, Writing – review & editing. ZXW: Supervision, Writing – review & editing. ZH: Resources, Supervision, Validation, Writing – review & editing. ZHW: Funding acquisition, Resources, Supervision, Validation, Writing – review & editing.
