# Supplementary material for: A case report of sustained remission after radiotherapy combined with ICI in NEPC with primary drug resistance to chemotherapy
Source: Front Oncol. 2024 Apr 26;14:1360956. doi: 10.3389/fonc.2024.1360956 (PMC11082353; doi:10.3389/fonc.2024.1360956)
Supplement: Supplementary file 1 [file DataSheet_1.docx]

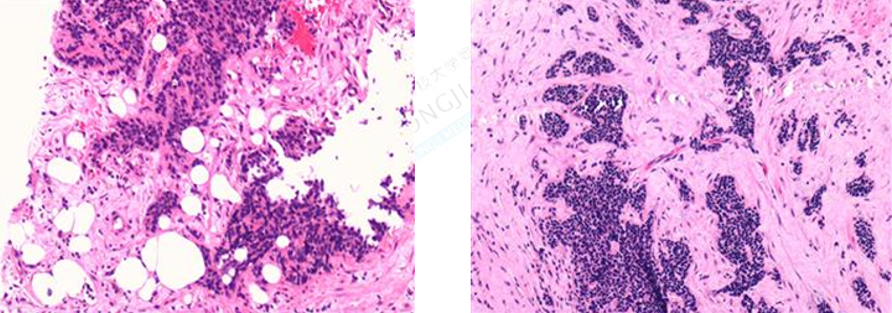


Suppl. Fig.

Pathological image of prostate biopsy on November 19. Professor Huang Jiaodi of the United States consulted pathology: poorly differentiated prostate cancer, considered small cell carcinoma with a small number of adenocarcinoma components. Professor Zhou Xiaojun consultation in Nanjing: highly malignant cancer tends to be poorly differentiated adenocarcinoma before treatment, but more like small cell carcinoma after treatment; small cell carcinoma transformation is not ruled out.
